# Matsuno, A., *et al.*, Molecular Morphology of Pituitary Cells, from Conventional Immunohistochemistry to Fluorescence Imaging. *Molecules* 2011, *16*, 3618-3635

**DOI:** 10.3390/molecules171011667

**Published:** 2012-09-28

**Authors:** Akira Matsuno, Akiko Mizutani, Hiroko Okinaga, Koji Takano, So Yamada, Shoko M. Yamada, Hiroshi Nakaguchi, Katsumi Hoya, Mineko Murakami, Masato Takeuchi, Mutsumi Sugaya, Johbu Itoh, Susumu Takekoshi, R. Yoshiyuki Osamura

**Affiliations:** 1 Department of Neurosurgery, Teikyo University Chiba Medical Center, Chiba 299-0111, Japan; Email: akiko@is.icc.u-tokai.ac.jp (A.M.); fwnt5053@nifty.com (S.Y.); merrityamada@hotmail.co.jp (S.M.Y.); hnakaguti@gmail.com (H.N.); khoya@med.teikyo-u.ac.jp (K.H.); muraminechan@yahoo.co.jp (M.M.); 2 Teikyo Heisei University, Tokyo 170-8445, Japan; Email: hhira-tky@umin.ac.jp; 3 Department of Nephrology and Endocrinology, University of Tokyo Hospital, Tokyo 113-0033, Japan; Email: ktakanotky@gmail.com; 4 Department of Rehabilitation, Teikyo University Chiba Medical Center, Chiba 299-0111, Japan; Email: akirama7@yahoo.co.jp (M.T.); m-sugaya@med.teikyo-u.ac.jp (M.S.); 5 Teaching and Research Support Center, Tokai University School of Medicine, Kanagawa 259-1100, Japan; Email: itohj@is.icc.u-tokai.ac.jp; 6 Department of Pathology, Tokai University School of Medicine, Kanagawa 259-1100, Japan; Email: takekos@is.icc.u-tokai.ac.jp; 7 Pathology Diagnosis Center, International University of Health and Welfare, Tokyo 108-8329, Japan; Email: osamura@iuhw.ac.jp

In the original manuscript, the word “fluorescein” was erroneously used indistinctly for “fluorescence” and “fluorescent”. Furthermore, “cyan fluorescent protein” was misspelled. These errors have been amended in an amended version of the manuscript, which is available from the Molecules website. The authors and publisher apologize for the inconvenience.

## Appendix

The corrected version can be accessed at: http://www.mdpi.com/1420-3049/17/9/11667/s1.
