# Supplementary material for: Molecular Morphology of Pituitary Cells, from Conventional Immunohistochemistry to Fluorescein Imaging
Source: Molecules. 2011 Apr 29;16(5):3618–35. doi: 10.3390/molecules16053618 (PMC6263291; doi:10.3390/molecules16053618)
Supplement: Supplementary File 1 [file molecules-16-03618-s001.pdf]

Molecules **2012**, *17*, 11667-11668; doi:10.3390/molecules171011667

OPEN ACCESS
molecules
ISSN 1420-3049
www.mdpi.com/journal/molecules

Correction

Matsuno, A., *et al.*, Molecular Morphology of Pituitary Cells, from Conventional Immunohistochemistry to Fluorescence Imaging. *Molecules* 2011, *16*, 3618-3635

Akira Matsuno [1,*], Akiko Mizutani [1,2], Hiroko Okinaga [2], Koji Takano [3], So Yamada [1], Shoko M. Yamada [1], Hiroshi Nakaguchi [1], Katsumi Hoya [1], Mineko Murakami [1], Masato Takeuchi [4], Mutsumi Sugaya [4], Johbu Itoh [5], Susumu Takekoshi [6] and R. Yoshiyuki Osamura [7]

[1] Department of Neurosurgery, Teikyo University Chiba Medical Center, Chiba 299-0111, Japan; E-Mails: akiko@is.icc.u-tokai.ac.jp (A.M.); fwnt5053@nifty.com (S.Y.); merrityamada@hotmail.co.jp (S.M.Y.); hnakaguti@gmail.com (H.N.); khoya@med.teikyo-u.ac.jp (K.H.); muraminechan@yahoo.co.jp (M.M.)
[2] Teikyo Heisei University, Tokyo 170-8445, Japan; E-Mail: hhira-tky@umin.ac.jp
[3] Department of Nephrology and Endocrinology, University of Tokyo Hospital, Tokyo 113-0033, Japan; E-Mail: ktakanotky@gmail.com
[4] Department of Rehabilitation, Teikyo University Chiba Medical Center, Chiba 299-0111, Japan; E-Mails: akirama7@yahoo.co.jp (M.T.); m-sugaya@med.teikyo-u.ac.jp (M.S.)
[5] Teaching and Research Support Center, Tokai University School of Medicine, Kanagawa 259-1100, Japan; E-Mail: itohj@is.icc.u-tokai.ac.jp
[6] Department of Pathology, Tokai University School of Medicine, Kanagawa 259-1100, Japan; E-Mail: takekos@is.icc.u-tokai.ac.jp
[7] Pathology Diagnosis Center, International University of Health and Welfare, Tokyo 108-8329, Japan; E-Mail: osamura@iuhw.ac.jp

* Author to whom correspondence should be addressed; E-Mail: akirakun@med.teikyo-u.ac.jp; Tel.: +81-436-62-1211; Fax: +81-436-62-1357.

Received: 11 September 2012; in revised form: 18 September 2012 / Accepted: 19 September 2012 / Published: 28 September 2012

In the original manuscript, the word "fluorescein" was erroneously used indistinctly for "fluorescence" and "fluorescent". Furthermore, "cyan fluorescent protein" was misspelled. These errors have been amended in an amended version of the manuscript, which is available from the Molecules website. The authors and publisher apologize for the inconvenience.

The corrected version can be accessed at: http://www.mdpi.com/1420-3049/17/9/11667/s1.

Reference

1. Matsuno, A.; Mizutani, A.; Okinaga, H.; Takano, K.; Yamada, S.; Yamada, S.M.; Nakaguchi, H.; Hoya, K.; Murakami, M.; Takeuchi, M.; *et al.* Molecular Morphology of Pituitary Cells, from Conventional Immunohistochemistry to Fluorescein Imaging. *Molecules* **2011**, *16*, 3618–3635.

© 2012 by the authors; licensee MDPI, Basel, Switzerland. This article is an open access article distributed under the terms and conditions of the Creative Commons Attribution license (http://creativecommons.org/licenses/by/3.0/).
