# Supplementary material for: Matsuno, A., et al., Molecular Morphology of Pituitary Cells, from Conventional Immunohistochemistry to Fluorescence Imaging. Molecules 2011, 16, 3618-3635
Source: Molecules. 2012 Sep 28;17(10):11667–8. doi: 10.3390/molecules171011667 (PMC6268085; doi:10.3390/molecules171011667)
Supplement: Supplementary file 1 [file molecules-17-11667-s001.pdf]

Molecules **2011**, *16*, 3618-3635; doi:10.3390/molecules16053618

OPEN ACCESS

molecules

ISSN 1420-3049

www.mdpi.com/journal/molecules

Review

Molecular Morphology of Pituitary Cells, from Conventional Immunohistochemistry to Fluorescence Imaging

Akira Matsuno [1,*], Akiko Mizutani [1,2], Hiroko Okinaga [2], Koji Takano [3], So Yamada [1], Shoko M. Yamada [1], Hiroshi Nakaguchi [1], Katsumi Hoya [1], Mineko Murakami [1], Masato Takeuchi [4], Mutsumi Sugaya [4], Johbu Itoh [5], Susumu Takekoshi [6] and R. Yoshiyuki Osamura [7]

[1] Department of Neurosurgery, Teikyo University Chiba Medical Center, Chiba 299-0111, Japan; E-Mails: akiko@is.icc.u-tokai.ac.jp (A.M.); fwnt5053@nifty.com (S.Y.); merrityamada@hotmail.co.jp (S.M.Y.); hnakaguti@gmail.com (H.N.); khoya@med.teikyo-u.ac.jp (K.H.); muraminechan@yahoo.co.jp (M.M.)

[2] Teikyo Heisei University, Tokyo 170-8445, Japan; E-Mail: hhira-tky@umin.ac.jp

[3] Department of Nephrology and Endocrinology, University of Tokyo Hospital, Tokyo 113-0033, Japan; E-Mail: ktakanotky@gmail.com

[4] Department of Rehabilitation, Teikyo University Chiba Medical Center, Chiba 299-0111, Japan; E-Mails: akirama7@yahoo.co.jp (M.T.); m-sugaya@med.teikyo-u.ac.jp

[5] Teaching and Research Support Center, Tokai University School of Medicine, Kanagawa 259-1100, Japan; E-Mail: itohj@is.icc.u-tokai.ac.jp

[6] Department of Pathology, Tokai University School of Medicine, Kanagawa 259-1100, Japan; E-Mail: takekos@is.icc.u-tokai.ac.jp

[7] Pathology Diagnosis Center, International University of Health and Welfare, Tokyo 108-8329, Japan; E-Mail: osamura@iuhw.ac.jp

* Author to whom correspondence should be addressed; E-Mail: akirakun@med.teikyo-u.ac.jp; Tel.: +81-436-62-1211; Fax: +81-436-62-1357.

Received: 9 March 2011; in revised form: 25 April 2011 / Accepted: 26 April 2011 / Published: 29 April 2011

Abstract: *In situ* hybridization (ISH) at the electron microscopic (EM) level is essential for elucidating the intracellular distribution and role of mRNA in protein synthesis. EM-ISH is considered to be an important tool for clarifying the intracellular localization of mRNA and the exact site of pituitary hormone synthesis on the rough endoplasmic reticulum. A combined ISH and immunohistochemistry (IHC) under EM (EM-ISH&IHC) approach has sufficient ultrastructural resolution, and provides two-dimensional images of the subcellular localization of pituitary hormone and its mRNA in a pituitary cell. The

advantages of semiconductor nanocrystals (quantum dots, Qdots) and confocal laser scanning microscopy (CLSM) enable us to obtain three-dimensional images of the subcellular localization of pituitary hormone and its mRNA. Both EM-ISH&IHC and ISH & IHC using Qdots and CLSM are useful for understanding the relationships between protein and mRNA simultaneously in two or three dimensions. CLSM observation of rab3B and SNARE proteins such as SNAP-25 and syntaxin has revealed that both rab3B and SNARE system proteins play important roles and work together as the exocytotic machinery in anterior pituitary cells. Another important issue is the intracellular transport and secretion of pituitary hormone. We have developed an experimental pituitary cell line, GH3 cell, which has growth hormone (GH) linked to enhanced yellow fluorescent protein (EYFP). This stable GH3 cell secretes GH linked to EYFP upon stimulation by Ca^{2+} influx or Ca^{2+} release from storage. This GH3 cell line is useful for the real-time visualization of the intracellular transport and secretion of GH. These three methods from conventional immunohistochemistry and fluorescence imaging allow us to consecutively visualize the process of transcription, translation, transport and secretion of anterior pituitary hormone.

Keywords: pituitary hormone; mRNA; intracellular transport and secretion; quantum dot; enhanced yellow fluorescent protein

---

1. Introduction

Molecular morphological research of production, transport and secretion of anterior pituitary hormones is essential for understanding the pathophysiology of pituitary cells. For this purpose, several molecular morphological studies are required. Immunoelectron microscopy has been developed for the observation of protein products and is now a sophisticated technique in the field of histopathology. *In situ* hybridization (ISH) at the electron microscopic (EM) level (EM-ISH) is another sophisticated technique that is essential for the intracellular identification of mRNA and the study of the role of mRNA in protein synthesis. We developed a non-radioisotopic EM-ISH method using biotinylated synthesized oligonucleotide probes, and applied this method to the ultrastructural visualization of growth hormone (GH) and prolactin (PRL) mRNAs and pathophysiological studies in rat pituitary cells [1–3]. In addition, we developed a combined EM-ISH and immunohistochemistry (IHC) (EM-ISH&IHC) technique for the purpose of simultaneously identifying pituitary hormone and its message in the same cell [4–6]. This method has been utilized to investigate the intracellular localization of pituitary hormone and its mRNA at the same time [4–11]. Confocal laser scanning microscopy (CLSM) enables us to observe subcellular organelles, mRNA and proteins three-dimensionally in routinely processed light microscopic specimens [12–23]. Semiconductor nanocrystals (quantum dots, Qdots) enable us to obtain multicolor images of molecules due to a narrow emission peak that can be excited via a single light wavelength [24,25]. In biological research, Qdots have been used for the detection of the signals of IHC and fluorescence *in situ* hybridization (FISH) [26–31]. Recently, the above-mentioned advantages of Qdots and CLSM have been successfully applied to three-dimensional imaging of the intracellular localization of mRNA and

protein [32,33]. Another important issue is the intracellular transport and secretion of pituitary hormone. We have investigated the modulations of the intracellular dynamics of growth hormone (GH), rab3B, soluble *N*-ethylmaleimide-sensitive factor attachment protein receptor (SNARE) proteins such as synaptosomal-associated protein of 25 kDa (SNAP-25) and syntaxin in rat pituitary cells, caused by growth hormone-releasing hormone (GHRH) and somatostatin (SRIF) [34,35]. In order to examine the relationships among molecules that play important roles in the transport and secretion of pituitary hormone, the real-time observation in a living cell is essential. Thus, we have developed an experimental pituitary cell line that has secretory granules of GH linked to enhanced yellow fluorescent protein (EYFP) [36]. The incorporation of rab3B in the secretion of GH through porosomes has been observed under CLSM [37]. In this review article, both EM-ISH&IHC using conventional immunohistochemistry and CLSM observation using fluorescence with Qdots and EYFP are discussed.

2. From Conventional Immunohistochemistry to Fluorescence Imaging

2.1. ISH at an Electron Microscopy Level

2.1.1. Preembedding ISH at an Electron Microscopy Level

As shown in our previous reports [1–3], hybridization signals for rat GH mRNA are demonstrated under light microscopy (LM) using avidin-biotin-complex (ABC) and horseradish peroxidase (HRP). EM-ISH with an antisense probe for rat GH mRNA reveals its diffuse localization on the polysomes of the entire rough endoplasmic reticulum (RER) (Figure 1). EM-ISH with a sense probe for GH mRNA shows negative signals.

Figure 1. (**a**) Photograph of preembedding EM-ISH for GH mRNA. GH mRNA is localized diffusely on the polysomes of the entire rough endoplasmic reticula (RER) (bar = 200 nm); (**b**) Control study with a sense probe for GH mRNA. GH mRNA is not localized on the RER (bar = 200 nm) [6].

(**a**) (**b**)

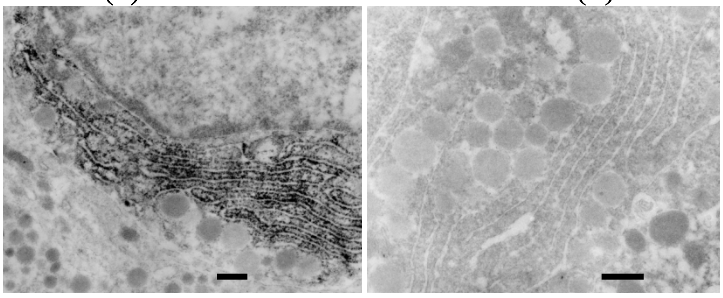

2.1.2. Postembedding ISH at an Electron Microscopic Level

As shown in our previous reports [1–3], hybridization signals for rat GH mRNA are localized on the polysomes of the RER using 20 nm streptavidin gold (Figure 2). EM-ISH with a sense probe for rat GH mRNA shows no hybridization signals. The hybridization signal intensity is lower than that observed with the preembedding method.

Figure 2. (**a**) Hybridization signals for rat GH mRNA are localized on the polysomes of the RER using 20 nm streptavidin gold (bar = 200 nm). The hybridization signal intensity is lower than that for the preembedding method; (**b**) EM-ISH with a sense probe for rat GH mRNA shows no hybridization signals (bar = 200 nm) [6].

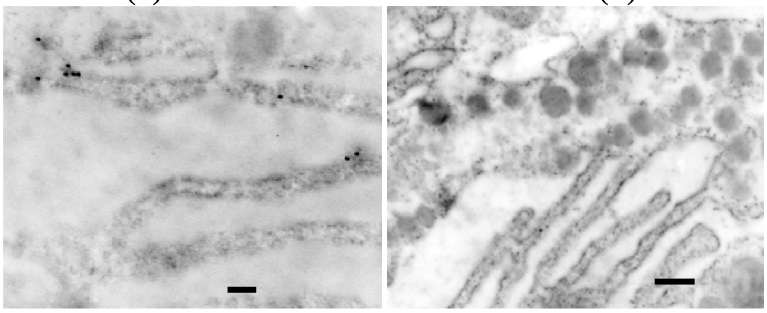

LM-ISH, which has become a widely used method for examining the tissue distribution and expression of mRNA, lacks the ultrastructural spatial relationship between mRNA and the encoded protein. This information, which can be obtained by EM-ISH, is needed to elucidate the intracellular distribution and role of mRNA for protein synthesis. The EM-ISH method was developed by several investigators [38–49], and was carried out in the different ways: preembedding [38,45,47,49], ultrathin frozen sections [43,44] and postembedding [39–41]. The major concerns of EM-ISH are how to maintain tissue morphology and retain the messages. Le Guellec *et al.* compared three EM-ISH methods: preembedding using 50 μm vibratome sections, using ultrathin frozen sections and postembedding using Lowicryl K4M-embedded specimens [42]. They stated that the ultrastructural preservation in EM-ISH using ultrathin frozen sections was poor, and that specimens embedded in Lowicryl K4M exhibited poorer ultrastructural preservation than those embedded in Epon resin [42]. Thus, in order to obtain satisfactory morphological preservation, we have routinely utilized the preembedding method for frozen sections fixed in 4% paraformaldehyde and the postembedding method for tissues embedded in LR White resin.

In the preembedding method, both ultrastructure and mRNA are sufficiently preserved. The preembedding method has a benefit in that hybridization signals can be confirmed with light microscopy. In the postembedding method using tissues embedded in LR White resin, ultrastructure is also sufficiently preserved. As described in our previous reports [1], compared with the preembedding method, the postembedding method has several drawbacks: (1) difficulty of message preservation during polymerization of LR White resin at high temperature for an extended period of time, which leads to mRNA degradation; (2) relatively high frequency of non-specific signals because of the non-specific affinity of gold particles. From these results, it is suggested that the preembedding method would be better and easier than the postembedding method because of its higher sensitivity and better preservation of mRNA.

2.2. Combined ISH and IHC at an Electron Microscopic Level

As shown in our previous reports [4–9], EM-ISH with an antisense probe for rat GH mRNA reveals its localization on the RER. Subsequent immunohistochemical staining using anti-rat GH antibody and

20 nm protein A colloidal gold identifies rat GH mainly on the secretory granules and also in the cisternae of the RER (Figure 3).

Figure 3. (**a**) EM-ISH with an antisense probe for rat GH mRNA reveals its localization on the rough endoplasmic reticulum (RER) as osmium black signals. Subsequent immunohistochemical staining using anti-rat GH antibody and 20 nm protein A colloidal gold particles (arrows) identifies rat GH mainly on the secretory granules and also in the cisternae of the RER (arrow heads) (bar = 200 nm, [6]); (**b**) Immunohistochemical control experiments reveal no positive reactions of protein A colloidal gold particles on the secretory granules, whereas positive signals for GH mRNA are identified as osmium black on the polysomes of the RER (bar = 200 nm, [6]).

(**a**) (**b**)

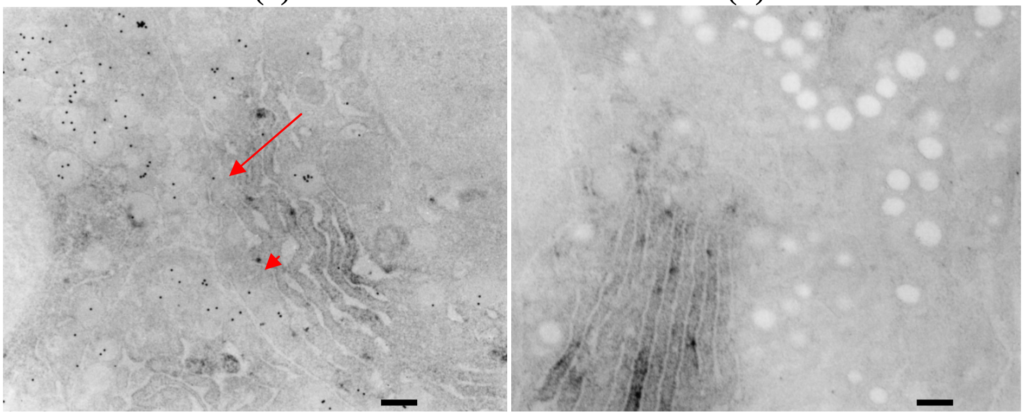

Another concern is how to visualize mRNA and encoded protein simultaneously in the same cell. There seem to be two major problems to be resolved in this ultrastructural double staining method. One is to retain the messages and the other is to maintain the immunoreactivity of the encoded protein in the same cell. Several reports describing the ultrastructural simultaneous detection of mRNA and encoded protein were published, in each of which the postembedding EM-ISH method using colloidal gold particle was utilized [46,50–53]. However, in these postembedding EM-ISH studies, the relatively frequent non-specific reactions of colloidal gold particles used for the detection of mRNA were observed in the cisternae of the RER. The EM-ISH method for Lowicryl K4M-embedded tissues is generally presumed to have some difficulty in morphological preservation. As for mRNA preservation, the preembedding EM-ISH method using frozen sections fixed in 4% paraformaldehyde has more advantages over the postembedding EM-ISH method using tissues embedded in LR White resin [1]. Frozen sections fixed in 4% paraformaldehyde have better morphological preservation than immediately frozen sections. Based on this assessment of maintaining tissue morphology and retaining the messages, we utilize the preembedding EM-ISH method using frozen sections fixed in 4% paraformaldehyde for the simultaneous detection of mRNA and encoded protein. In general, osmification and embedment in Epon resin are reported to decrease the immunoreactivity of the targeted protein. However, it is shown in this study that immunoreactivity can be retrieved by the etching process using H_2O_2 or sodium periodate even after modification such as osmification and embedment in Epon resin, and that tissues embedded in Epon resin can be used for the ultrastructural simultaneous detection of messages and encoded proteins. The only problem is the deosmification and

degradation of the signals of mRNA, which is caused by the etching process using H_2O_2 or sodium periodate. In order to resolve this problem, we have used LR White resin for tissue embedment [5]. In LR White resin-embedded tissues, retrieval of immunoreactivity using H_2O_2 or sodium periodate is not required, and therefore the gradation of the signals of mRNA can be avoided. Translation of mRNA for secreted proteins such as GH is initiated on free ribosomes and then translocated to the polysomes on the RER with the aid of signal recognition particles once the signal peptide is produced. Synthesized proteins are secreted into the luminal space of the RER and subsequently transported to secretory granules via Golgi's apparatus. The ultrastructural double staining method for mRNA and encoded protein is considered to provide an important clue for elucidating the intracellular spatial relationship of mRNA translation and translated protein. The EM-ISH&IHC method has high ultrastructural resolution, and can provide two-dimensional images of the intracellular localization of mRNA and protein. Although its images are two-dimensional the EM-ISH&IHC method is essential for high resolution analyses of subcellular molecules and organelles. Other important molecular morphological methods include combinations with fluorescence techniques. Christian *et al.* exploited a combination of fluorescence-activated cell analysis/sorting and EM to detect, characterize and localize LC1-binding sites on the surface of dispersed rat anterior pituitary cells [54]. Harper *et al.* used live-cell imaging to quantify patterns of reporter gene expression in dispersed lactotrophic cells or intact pituitary tissue from bacterial artificial chromosome transgenic rats in which a large PRL genomic fragment directed expression of luciferase or destabilised enhanced green fluorescent protein (d2EGFP) [55]. This method has an advantage of possible 4-dimensional detection in time course observation.

2.3. Combined ISH and IHC Using Qdots for the Detection of mRNA and Protein

With the confocal mode, GH protein was observed as a 655 nm emission signal using using Qdot 655 conjugated with anti-rabbit IgG, and GH mRNA was observed as a 605 nm emission signal using Qdot 605 conjugated with streptavidin (Quantum Dot Corp., Hayward, CA, USA). When GH mRNA and protein were located either in the same or adjacent places, their signals were detected in the mixed color image (Figure 4).

Figure 4. CLSM imaging of GH protein at 655 nm emission with Qdot 655 (red), and GH mRNA at 605 nm emission using Qdot 605 (green). When GH mRNA and protein were located in the same or adjacent places, their signals were detected in the mixed color images (yellow) (stereo-images, bar = 2 μm [33]).

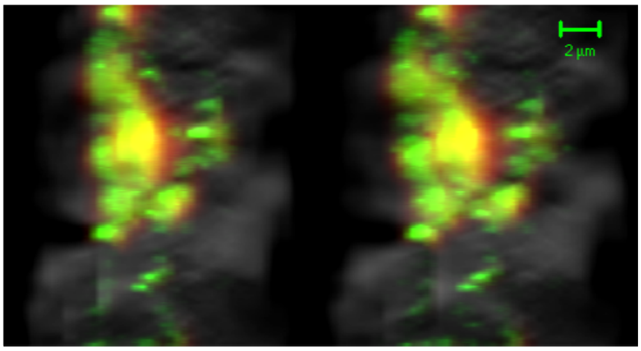

Qdots have narrow, symmetric emission spectra with multiple resolvable colors that can be excited simultaneously using a single excitation wavelength. The color of Qdots can be tuned to any chosen wavelength by simply changing their size. This property is capable of multiple labeling of subcellular molecules. Qdots have an advantage over conventional fluorophores, such as FITC and Texas Red, due to their photostability and prominent signal intensity. Qdot signals were more than 11-fold stronger than those of fluorescein [31]. These properties enable us to visualize the intracellular localization of pituitary hormone and mRNA by using different sized Qdots with CLSM [32,33]. This analysis has several merits: (i) it can be used with light microscopic specimens; (ii) can be observed in any chosen cells and any chosen depth of the section; and (iii) allows the reconstruction of three-dimensional images. Three-dimensional images of the intracellular localization of mRNA and encoded protein enhance our three-dimensional understanding concerning the localization of mRNA and secreted protein. Two different sized Qdots can discriminate between two molecules that are located in the three-dimensional distance more than 25 ± 13 nm [56,57].

ISH and IHC with Qdots and CLSM are capable of the simultaneous and three-dimensional visualization of the relationship between protein and mRNA, whereas EM-ISH&IHC method enables us to visualize the two-dimensional relationship between protein and mRNA simultaneously in high resolutions. Both EM-ISH&IHC and ISH & IHC using Qdots and CLSM are useful for understanding the relationship between protein and mRNA simultaneously in two or three dimensions.

2.4. Functional Analyses of rab3B in Pituitary Cells and the SNARE System and rab3B in Pituitary Cells

CLSM of immunohistochemical double stainings for SNAP-25, syntaxin and rab3B demonstrated co-localization of rab3B and these SNARE proteins in GHRH-treated rats, and their dissociation in SRIF-treated rats (Figure 5). These results suggest that rab3B plays a principal role in GH secretion from the anterior pituitary cells and that SNAP-25 and syntaxin act in association with rab3B in functional regulation of GH secretion.

Low molecular weight GTP-binding proteins of the rab family act as the central regulators of vesicular traffic. The rab3 subfamily (rab3A, B, C and D) proteins are associated with membrane vesicles or granules that are undergoing exocytotic fusion with the plasma membrane [58,59]. High expression of rab3A is observed in brain tissue [60], whereas rab3B is the major form found in the anterior pituitary [59]. Lledo *et al.* reported that rab3B is a key intracellular signaling molecule that can control exocytosis in anterior pituitary cells [59]. Tasaka *et al.* suggested that rab3B is indispensable for gonadotropin-releasing hormone (GnRH)-induced gonadotropin release [61]. They reported that rab3B is involved in basal and GnRH-induced gonadotropin release, and that rab3B is essential for GnRH-regulated exocytosis downstream of cytosolic Ca^{2+} in gonadotrophs. Tahara *et al.* immunohistochemically investigated the localization of rab3 in five human non-tumorous pituitaries and 114 human pituitary adenomas, finding that rab3 is preferentially expressed in GH-secreting cells in non-tumorous pituitaries [62]. They concluded that rab3 might be involved in regulating exocytosis of secretory granules from anterior pituitary cells, particularly GH-secreting cells, which display characteristic densely granulated cytological features.

Figure 5. Confocal laser scanning microscopic observation of immunohistochemical double staining of rab3B (confocal mode; green), SNAP-25 (transmittance mode; black): Signals of SNAP-25 are noted in non-treated control rats (**a**); GHRH-treated rats (**b**); and SRIF-treated rats (**c**); When signals of rab3B are combined in the same cells, compared with non-treated control rats (**d**); the co-localization of rab3B and SNAP-25 is noted in GHRH-treated rats (**e**); whereas the dissociation of rab3B and SNAP-25 is noted in SRIF-treated rats (**f**); Confocal laser scanning microscopic observation of immunohistochemical double staining of rab3B (confocal mode; green), syntaxin (transmittance mode; black): Signals of syntaxin are noted in non-treated control rats (**g**); GHRH-treated rats (**h**); and SRIF-treated rats (**i**); When signals of rab3B are combined in the same cells, compared with non-treated control rats (**j**); the co-localization of rab3B and syntaxin is noted in GHRH-treated rats (**k**); whereas the dissociation of rab3B and syntaxin is noted in SRIF-treated rats (**l**) (bar = 5 μm, [35]).

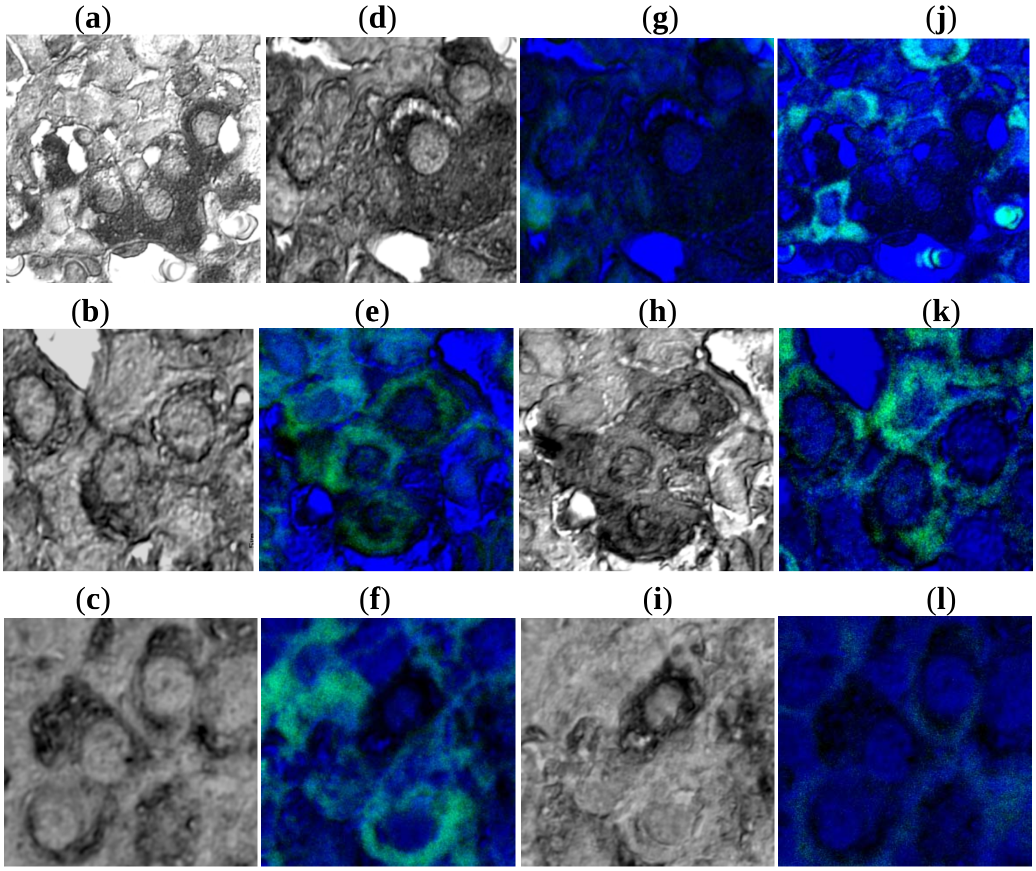

We demonstrated the close relationship between GH secretion and rab3B under CLSM [34,35]. In rat pituitary cells treated with GHRH, one of the major stimulatory factors of GH production and secretion, increased rab3B immunoreactivity and decreased GH immunoreactivity are apparent. In rat pituitary cells treated with SRIF, one of the major inhibitory factors of GH production and secretion, decreased rab3B immunoreactivity and increased GH immunoreactivity can be noted. These results strongly suggest a principal role for rab3B in GH secretion from the anterior pituitary [34,35].

The SNARE system comprises numerous related proteins. Among them, target-SNARE (t-SNARE) proteins include SNAP-25 and syntaxin, whereas vesicle-SNARE (v-SNARE) proteins include

vesicle-associated membrane protein-2 (VAMP-2), which is localized on the membrane of secretory granules. Synaptic proteins localized to the synaptic vesicle membrane include synaptotagmin, VAMP, cysteine string protein (CSP), SV2, rabphilin, rab3, synaptophysin, and synapsin. Synaptic proteins that are found within or at the plasma membrane include SNAP-25, syntaxin, and a 35-kDa protein. SNAP-25 is attached to the plasma membrane through fatty acylated cysteine residues [63,64]. Syntaxin is a membrane-associated protein that binds to synaptotagmin [65], VAMP [66], SNAP-25 [67], *N*-ethylmaleimide sensitive factor (NSF) [67], soluble NSF-attachment proteins (SNAPs) [67,68], and neural-specific syntaxin-binding protein (n-Sec1) [69–71]. Exocytotic proteins with a cytoplasmic location that can associate with these vesicle or plasma membrane proteins include SNAPs, NSF and mammalian homologue of unc-18 (munc-18). SNAPs are essential for membrane fusion [67]. Munc-18 is closely associated with syntaxin, and to interfere with the binding of VAMP and SNAP-25 to syntaxin [72]. The exact roles of these proteins with regard to exocytotic mechanisms in the pituitary gland remain to be elucidated. Limited information on the presence and the cellular localization of SNARE proteins have been presented for anterior pituitary cells. As for the membrane fusion of secretory granules in anterior pituitary cells, the SNARE mechanism is essential.

Jacobsson *et al.* studied the expression and cellular localization, in rat pituitary gland, of several protein components that are essential for exocytotic membrane fusion in neurons [73]. They applied ISH and immunohistochemical techniques to the detection of proteins such as SNAP-25, syntaxin, VAMP-2, synaptotagmin, CSP, cellubrevin, and munc-18, showing the presence of several protein components and their isoform-specific mRNAs in rat pituitary. They suggested that these proteins, similar to their roles in the regulation of synaptic neurotransmitter release, might participate in exocytotic events in pituitary endocrine cells. Salinas *et al.* investigated the presence of syntaxin-1 and SNAP-25 in different parts of mouse, guinea pig and cat pituitaries using immunohistochemical methods, and suggested that syntaxin-1 and SNAP-25 are involved in the hormonal secretory process of both the adenohypophysis and neurohypophysis in these species [74]. Quintanar *et al.* analyzed the expression of SNAP-25 and syntaxin-1 in the adenohypophyses of hypothyroid rats, and found that thyroidectomy resulted in changes to both expression and immunoreactivity of SNAP-25 and syntaxin-1, and that these effects could be reversed by T4 administration [75]. However, the direct function of rab3B in conjunction with the secretion of pituitary hormones and SNARE system remains unclear. Elucidation of the role of rab3B with regard to SNARE mechanisms represents an important issue.

We therefore undertook experiments to elucidate the role of rab3B in GH secretion and the mutual relationships with SNARE proteins such as SNAP-25 and syntaxin [34,35]. Both rab3B and SNARE system proteins play important roles and work together as the exocytotic machinery in anterior pituitary cells.

2.5. Intracellular Transport and Secretion of EYFP-GH and Synergistic Dynamics of rab3B and GH in Porosome

In order to investigate, in real time, the transport and secretion of pituitary hormone, we developed a stable experimental pituitary cell line, GH3 cell, which has secretory granules of GH linked to EYFP [36]. The GH3 cells transfected with pCMV- sig- EYFP-GH-1 were incubated with culture medium of high K 60 mEq/L concentration, and they were observed under CLSM, which showed that granules of GH

were secreted. Control experiment with no treatment showed no secretion of secretory granules. To investigate the synergistic dynamics of rab3B and GH in porosome, we have constructed rat rab3B-enhanced cyan fluorescent protein (ECFP) plasmid for transfection of GH3 cell [37] (Figure 6). A cDNA fragment encoding rat rab3B was amplified by RT-PCR and cloned in-frame into the EcoRI/BamHI sites of pECFP-N1 (Clontech Laboratories, Inc. Mountain View, CA, USA), which expresses rab3B-ECFP fusion proteins in the mammalian cell. Plasmids were purified using Qiagen midi-prep kit (Qiagen GmbH, Hilden, Germany), and were verified by nucleotide sequencing. Transfection of GH3 cells was performed using lipofectamine 2000 (Invitrogen Corp., Carlsbad, CA, USA). Cells were observed under CLSM for the inspection of the dynamics of EYFP-GH and ECFP-rab3B.

Figure 6. When GH3 cells transfected with pCMV- sig- EYFP-GH-1 were incubated with culture medium of high K 60 mEq/L concentration, CLSM observation revealed that granules of GH were secreted (**a**: 0 min., before treatment, **b**: 10 min. after high K treatment [36]).

(**a**) (**b**)

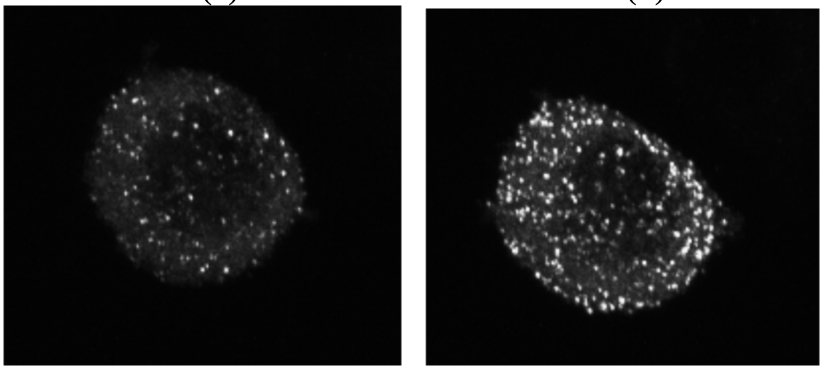

Green fluorescent protein (GFP) has been used widely in cell biology for the real-time visualization of cellular processes [76]. GFP has been used as a maker that can be attached to proteins without alterations in its fluorescence. The function of the protein itself is usually unchanged. The GFP linked to protein allows us to trace, in real time, the intracellular distribution, transport and secretion of the molecules. In the field of pituitary hormone research, there is few reports that describe the establishment of transgene expressing pituitary hormone linked to GFP. It is critically important to produce a sequence that would target itself to the right cell types, but not alter the physiology of the cells themselves. The signal peptide is critical to allow entry into the rough endoplasmic reticulum. Recently, Magoulas *et al.* produced a construct encoding the gene for enhanced GFP (EGFP) linked to sequences of human GH [77]. They used two different lengths of the 5-coding sequence of the GH gene fused with EGFP: One had the longer sequence corresponding to the first 48 amino acids of GH, and the other had the shorter sequence corresponding to the first eight amino acids of the GH signal peptide. They transfected both constructs into GC cell line that produces GH normally. They used the longer version of the EGFP-GH construct to generate transgenic mice expressing EGFP linked to GH sequences. However, there were no reports that described the establishment of a stable cell line expressing pituitary hormone linked to GFP. Then, in order to investigate, in real time, the transport and secretion of pituitary hormone, we developed a stable experimental pituitary cell line, GH3 cell, which has secretory granules of GH linked to EYFP [36]. This GH3 cell has secretory granules of GH

linked to EYFP, and secretes this molecule upon stimulated by Ca^{2+} influx or Ca^{2+} release from storage. There is an experimental limitation when using the GH3 cell line, because GH3 cells lack GHRH receptors. Lee *et al.* restored GHRH responsiveness in GH3 cells by adenoviral vectors to transfer the human GHRH receptor to GH3 cells [78]. Another issue is that experimental cell lines have an altered transcriptome and their physiological relevance is limited. Nevertheless, these GH3 cells are useful for the real-time visualization of the intracellular transport and secretion of GH. Moreover, using the experimental cell line expressing EYFP-GH and ECFP-rab3B, we have demonstrated the role of rab3B in the vesicular budding and GH secretion through porosome. These bioimagings of rab3B support the role of rab3B in cooperation of GH secretion with SNARE system and porosome.

Additionally it is noteworthy that He *et al.* developed a system to use secreted fluorescent proteins as surrogate markers for the continuous on-line monitoring of hormone release from perfused tissue slices [79]. They tested this system using GH-GFP transgenic rats with GFP targeted to the secretory vesicles of pituitary GH cells. This method provides useful insight into the release kinetics from large populations of pituitary cells, and fills a temporo-spatial gap between single vesicle and single cell monitoring of exocytosis in milliseconds, and *in vivo* sampling studies of release into the bloodstream on a time scale of minutes.

3. Overview of Experimental Procedures

3.1. ISH at an Electron Microscopic Level

3.1.1. Preembedding ISH at an Electron Microscopic Level

The details of the EM-ISH&IHC method are described in our previous reports [1–3]. Briefly, the anterior lobes of rat pituitary gland were fixed in 4% paraformaldehyde dissolved in 0.01 M phosphate buffered saline pH 7.4 (PBS). Hybridization was carried out using biotinylated oligonucleotide probes. The hybridized signals of mRNA were detected with ABC-HRP, and then developed with diaminobenzidine (DAB) and H_2O_2. After osmification and dehydration with ethanol, tissue sections were embedded in Epon resin. After polymerization, ultrathin sections were inspected under electron microscopy. The control experiments carried out are hybridization with probes of sense or scramble sequence and without probes.

3.1.2. Postembedding ISH at an Electron Microscopic Level

Briefly, in this method the anterior lobes of rat pituitary gland were fixed in 4% paraformaldehyde dissolved in 0.01 M phosphate buffered saline pH 7.4 (PBS). After fixation, tissues were embedded in LR White resin (Polyscience, Warrington, PA, USA). Ultrathin sections were retrieved on nickel grids. Hybridization was carried out on the grids, and the hybridization signals are developed with 20 nm streptavidin gold (British Biocell International, Cardiff, UK). The grids were inspected under electron microscopy. The control experiments carried out are hybridization with probes of sense or scramble sequence and without probes.

3.2. Combined ISH and IHC at an Electron Microscopy Level

Briefly, in these experiments hybridization was carried out using biotinylated oligonucleotide probes. The hybridized signals of mRNA were detected with streptavidin-biotin complex and horseradish peroxidase, and then developed with diaminobenzidine (DAB) and H_2O_2. After osmification and dehydration with ethanol, tissue sections were embedded in Epon resin. After polymerization, ultrathin sections were attached to nickel grids. Subsequently, the immunoreactivity of the targeted protein was retrieved by 10% H_2O_2 or with 4% sodium periodate. Immunohistochemical staining was carried out using primary antibody, and the immunoreaction was visualized with 20 nm protein A colloidal gold. The ultrathin sections were inspected under electron microscopy.

3.3. Combined ISH and IHC Using Qdots for the Detection of mRNA and Protein

The detailed method was described in our previous report [32]. Hybridization was carried out using biotinylated oligonucleotide probes, and subsequently, immunohistochemical staining was carried out using primary antibody. Both the hybridized signals of mRNA and the immunopositive reactions were detected with different sized Qdots (Quantum Dot Corp.) that had different emission peaks. The tissue sections were inspected under CLSM.

3.4. Functional Analyses of rab3B in Pituitary Cells and the SNARE System and rab3b in Pituitary Cells

The detailed method was described in our previous report [34,35]. Briefly, the pituitary glands were removed from GHRH-injected rats, SRIF-injected rats, and untreated control rats. Routinely processed paraffin sections were used for light microscopic immunohistochemical double staining for rab3B and SNAP-25, and for rab3B and syntaxin. For subcellular analyses of SNAP-25, syntaxin and rab3B, the tissue sections were examined under CLSM. The immunoreactions for SNAP-25 and syntaxin developed by DAB were observed under CLSM with the transmittance mode, and rab3B immunoreactions developed by alkaline phosphatase (ALP)-Fuchsin were observed under CLSM with the confocal mode.

3.5. Intracellular Transport and Secretion of EYFP-GH and Synergistic Dynamics of rab3B and GH in Porosome

The details of the construct of EYFP-GH were and establishment of stable GH3 cell expressing described in our previous report [36]. The GH-EYFP fusion construct pCMV-Sig- EYFP-GH-1 was derived from pEYFP-N1 (Clontech Laboratories, Inc.) and contained a sequence encoding the rat GH signal peptide (1 to 26 in the rat GH amino-acid sequence) and the EYFP-coding segment, followed by another rat GH coding sequence (27 to 217 in the rat GH amino-acid sequence). GH3 cells were transfected with plasmid DNA in low serum Opti-MEM using lipofectamine 2000 (Invitrogen Corp.) for 4–5 h. The GH3 cell transfected with pCMV- sig- EYFP-GH-1 had secretory granules that emitted yellow color in the cytoplasm. The expression of EYFP-GH protein was confirmed by western blotting as described in our previous report [36]. Cells were incubated with culture medium of high K 60 mEq/L concentration and observed under CLSM.

To investigate the synergistic dynamics of rab3B and GH in the porosome, we have constructed rat rab3B-enhanced cyan fluorescent protein (ECFP) plasmid for transfection of GH3 cell [37]. A cDNA fragment encoding rat rab3B was amplified by RT-PCR and cloned in-frame into the EcoRI/BamHI sites of pECFP-N1 (Clontech Laboratories, Inc.), which expresses rab3B-ECFP fusion proteins in the mammalian cell. Plasmids were purified using Qiagen midi-prep kit (Qiagen GmbH), and were verified by nucleotide sequencing. Transfection of GH3 cells was performed using lipofectamine 2000 (Invitrogen Corp.). Cells were observed under CLSM for the inspection of the dynamics of EYFP-GH and ECFP-rab3B.

4. Conclusions

For two- or three- dimensional imagings of subcellular localization of pituitary hormone and its mRNA, both EM-ISH&IHC and CLSM observation using Qdots are essential. CLSM observation of rab3B and SNARE proteins such as SNAP-25 and syntaxin revealed that both rab3B and SNARE system proteins play important roles and work together as the exocytotic machinery in anterior pituitary cells. Experimental cell line transfected with EYFP-linked pituitary hormone is very useful for the inspection of intracellular transport and secretion of pituitary hormone. These three methods from conventional immunohistochemistry and fluorescence imaging enable us to visualize consecutively the process of transcription, translation, transport, and secretion of anterior pituitary hormone.

References

1. Matsuno, A.; Ohsugi, Y.; Utsunomiya, H.; Takekoshi, S.; Osamura, R.Y.; Watanabe, K.; Teramoto, A. Ultrastructural distribution of growth hormone, prolactin mRNA in normal rat pituitary cells: A comparison between preembedding and postembedding methods. *Histochemistry* **1994**, *102*, 265–270.
2. Matsuno, A.; Teramoto, A.; Takekoshi, S.; Utsunomiya, H.; Ohsugi, Y.; Kishikawa, S.; Osamura, R.Y.; Kirino, T.; Lloyd, R.V. Application of biotinylated oligonucleotide probes to the detection of pituitary hormone mRNA using Northern blot analysis, *in situ* hybridization at light and electron microscopic levels. *Histochem. J.* **1994**, *26*, 771–777.
3. Matsuno, A.; Ohsugi, Y.; Utsunomiya, H.; Takekoshi, S.; Sanno, N.; Osamura, R.Y.; Watanabe, K.; Teramoto, A.; Kirino, T. Changes in the ultrastructural distribution of prolactin and growth hormone mRNAs in pituitary cells of female rats after estrogen and bromocriptine treatment, studied using *in situ* hybridization with biotinylated oligonucleotide probes. *Histochem. Cell Biol.* **1995**, *104*, 37–45.
4. Matsuno, A.; Utsunomiya, H.; Ohsugi, Y.; Takekoshi, S.; Sanno, N.; Osamura, R.Y.; Nagao, K.; Tamura, A.; Nagashima, T. Simultaneous ultrastructural identification of growth hormone and its messenger ribonucleic acid using combined immunohistochemistry and non-radioisotopic *in situ* hybridization: A technical note. *Histochem. J.* **1996**, *28*, 703–707.
5. Matsuno, A.; Ohsugi, Y.; Utsunomiya, H.; Takekoshi, S.; Munakata, S.; Nagao, K.; Osamura, R.Y.; Tamura, A.; Nagashima, T. An improved ultrastructural double-staining method of rat growth hormone and its mRNA using LR White resin: A technical note. *Histochem. J.* **1998**, *30*, 105–109.

6. Matsuno, A.; Nagashima, T.; Osamura, R.Y.; Watanabe, K. Application of ultrastructural *in situ* hybridization combined with immunohistochemistry to pathophysiological studies of pituitary cell: Technical review. *Acta Histochem. Cytochem.* **1998**, *31*, 259–265.
7. Matsuno, A.; Nagashima, T.; Takekoshi, S.; Utsunomiya, H.; Sanno, N.; Osamura, R.Y.; Watanabe, K.; Tamura, A.; Teramoto, A. Ultrastructural simultaneous identification of growth hormone and its messenger ribonucleic acid. *Endocr. J.* **1998**, *45*, S101–S104.
8. Matsuno, A.; Itoh, J.; Osamura, R.Y.; Watanabe, K.; Nagashima, T. Electron microscopic and confocal laser scanning microscopic observation of subcellular organelles and pituitary hormone mRNA: Application of ultrastructural *in situ* hybridization and immunohistochemistry to the pathophysiological studies of pituitary cells. *Endocr. Pathol.* **1999**, *10*, 199–211.
9. Matsuno, A.; Nagashima, T.; Ohsugi, Y.; Utsunomiya, H.; Takekoshi, S.; Munakata, S.; Nagao, K.; Osamura, R.Y.; Watanabe, K. Electron microscopic observation of intracellular expression of mRNA and its protein product: Technical review on ultrastructural *in situ* hybridization and its combination with immunohistochemistry. *Histol. Histopathol.* **2000**, *15*, 261-268.
10. Osamura, R.Y.; Itoh, Y.; Matsuno, A. Application of plastic embedding to electron microscopic immunocytochemistry and *in situ* hybridization in observations of production and secretion of peptide hormones. *J. Histochem. Cytochem.* **2000**, *48*, 885–892.
11. Osamura, R.Y.; Tahara, S.; Kurotani, R.; Sanno, N.; Matsuno, A.; Teramoto, A. Contributions of immunohistochemistry and *in situ* hybridization to the functional analysis of pituitary adenomas. *J. Histochem. Cytochem.* **2000**, *48*, 445–458.
12. Arndt-Jovin, D.J.; Robert-Nicoud, M.; Kaufman, S.J.; Jovin, T.M. Fluorescence digital imaging microscopy in cell biology. *Science* **1985**, *230*, 247–256.
13. Arndt-Jovin, D.J.; Robert-Nicoud, M.; Jovin, T.M. Probing DNA structure and function with a multi-wavelength fluorescence confocal laser microscope. *J. Microsc.* **1990**, *157*, 61-72.
14. Bauman, J.G.; Bayer, J.A.; van Dekken, H. Fluorescent in-situ hybridization to detect cellular RNA by flow cytometry and confocal microscopy. *J. Microsc.* **1990**, *157*, 73–81.
15. Hozak, P.; Novak, J.T.; Smetana, K. Three-dimensional reconstructions of nucleolus-organizing regions in PHA-stimulated human lymphocytes. *Biol. Cell* **1989**, *66*, 225–233.
16. Itoh, J.; Osamura, R.Y.; Watanabe, K. Subcellular visualization of light microscopic specimens by laser scanning microscopy and computer analysis: A new application of image analysis. *J. Histochem. Cytochem.* **1992**, *40*, 955–967.
17. Itoh, J.; Sanno, N.; Matsuno, A.; Itoh, Y.; Watanabe, K.; Osamura, R.Y. Application of confocal laser scanning microscopy (CLSM) to visualize prolactin (PRL) and PRL mRNA in the normal and estrogen-treated rat pituitary glands using non-fluorescent probes. *Microsc. Res. Tech.* **1997**, *39*, 157-167.
18. Itoh, J.; Matsuno, A.; Yamamoto, Y.; Kawai, K.; Serizawa, A.; Watanabe, K.; Itoh, Y.; Osamura, R.Y. Confocal laser scanning microscopic imaging of subcellular organelles, mRNA, protein products, and the microvessel environment. *Acta Histochem. Cytochem.* **2001**, *34*, 285–297.
19. Michel, E.; Parsons, J.A. Histochemical and immunocytochemical localization of prolactin receptors on Nb2 lymphoma cells: Applications of confocal microscopy. *J. Histochem. Cytochem.* **1990**, *38*, 965–973.

20. Robinson, J.M.; Batten, B.E. Detection of diaminobenzidine reactions using scanning laser confocal reflectance microscopy. *J. Histochem. Cytochem.* **1989**, *37*, 1761–1765.
21. Takamatsu, T.; Fujita, S. Microscopic tomography by laser scanning microscopy and its three-dimensional reconstruction. *J. Microsc.* **1988**, *149*, 167–174.
22. Tao, W.; Walter, R.J.; Berns, M.W. Laser-transected microtubules exhibit individuality of regrowth, however most free new ends of the microtubules are stable. *J. Cell. Biol.* **1988**, *107*, 1025–1035.
23. White, J.G.; Amos, W.B.; Fordham, M. An evaluation of confocal versus conventional imaging of biological structures by fluorescence light microscopy. *J. Cell Biol.* **1987**, *105*, 41–48.
24. Bruchez, M., Jr.; Moronne, M.; Gin, P.; Weiss, S.; Alivisatos, A.P. Semiconductor nanocrystals as fluorescent biological labels. *Science* **1998**, *281*, 2013–2016.
25. Wang, C.; Shim, M.; Guyot-Sionnest, P. Electrochromic nanocrystal quantum dots. *Science* **2001**, *291*, 2390–2392.
26. Chan, W.C.; Maxwell, D.J.; Gao, X.; Bailey, R.E.; Han, M.; Nie, S. Luminescent quantum dots for multiplexed biological detection and imaging. *Curr. Opin. Biotechnol.* **2002**, *13*, 40–46.
27. Gao, X.; Chan, W.C.; Nie, S. Quantum-dot nanocrystals for ultrasensitive biological labeling and multicolor optical encoding. *J. Biomed. Opt.* **2002**, *7*, 532–537.
28. Gao, X.; Nie, S. Molecular profiling of single cells and tissue specimens with quantum dots. *Trends Biotechnol.* **2003**, *21*, 371–373.
29. Han, M.; Gao, X.; Su, J.Z.; Nie, S. Quantum-dot-tagged microbeads for multiplexed optical coding of biomolecules. *Nat. Biotechnol.* **2001**, *19*, 631–635.
30. Pathak, S.; Choi, S.K.; Arnheim, N.; Thompson, M.E. Hydroxylated quantum dots as luminescent probes for *in situ* hybridization. *J. Am. Chem. Soc.* **2001**, *123*, 4103–4104.
31. Xiao, Y.; Barker, P.E. Semiconductor nanocrystal probes for human metaphase chromosomes. *Nucl. Acid. Res*. **2004**, *32*, e28.
32. Matsuno, A.; Itoh, J.; Takekoshi, S.; Nagashima, T.; Osamura, R.Y. Three-dimensional imagings of the intracellular localization of growth hormone and prolactin and their mRNA using nanocrystal (Quantum dot) and confocal laser scanning microscopy techniques. *J. Histochem. Cytochem.* **2005**, *53*, 833–838.
33. Matsuno, A.; Itoh, J.; Takekoshi, S.; Nagashima, T.; Osamura, R.Y. Two- or three- dimensional imagings of simultaneous visualization of rat pituitary hormone and its mRNA, comparison between electron microscopy and confocal laser scanning microscopy with semiconductor nanocrystals (Quantum dots). *Acta Histochem. Cytochem.* **2005**, *38*, 253–256.
34. Matsuno, A.; Itoh, J.; Takekoshi, S.; Itoh, Y.; Ohsugi, Y.; Katayama, H.; Nagashima, T.; Osamura, R.Y. Dynamics of subcellular organelles, growth hormone, rab3b, SNAP-25, and syntaxin in rat pituitary cells caused by growth hormone releasing hormone and somatostatin. *Microsc. Res. Tech.* **2003**, *62*, 232–239.
35. Matsuno, A.; Itoh, J.; Takekoshi, S.; Nagashima, T.; Osamura, R.Y. Functional and morphological analyses of rab proteins and the soluble *N*-ethylmaleimide-sensitive factor attachment protein receptor (SNARE) system in the secretion of pituitary hormones. *Acta Histochem. Cytochem.* **2003**, *36*, 501–506.

36. Matsuno, A.; Mizutani, A.; Itoh, J.; Takekoshi, S.; Nagashima, T.; Okinaga, H.; Takano, K.; Osamura, R.Y. Establishment of stable GH3 cell line expressing enhanced yellow fluorescein protein-growth hormone fusion protein. *J. Histochem. Cytochem.* **2005**, *53*, 1177–1180.
37. Matsuno, A.; Itoh, J.; Mizutani, A.; Takekoshi, S.; Osamura, R.Y.; Okinaga, H.; Ide, F.; Miyawaki, S.; Uno, T.; Asano, S.; *et al.* Co-transfection of EYFP-GH and ECFP-rab3B in an experimental pituitary GH3 cell: A role of rab3B in secretion of GH through porosome. *Folia Histochem. Cytobiol.* **2008**, *46*, 419–421.
38. Guitteny, A.F.; Bloch, B. Ultrastructural detection of the vasopressin messenger RNA in the normal and Brattleboro rat. *Histochemistry* **1989**, *92*, 277–281.
39. Jirikowski, G.F.; Sanna, P.P.; Bloom, F.E. mRNA coding for oxytocin is present in axons of the hypothalamo-neurohypophyseal tract. *Proc. Natl. Acad. Sci. USA* **1990**, *87*, 7400–7404.
40. Le Guellec, D.; Frappart, L.; Willems, R. Ultrastructural localization of fibronectin mRNA in chick embryo by *in situ* hybridization using 35S or biotin labeled cDNA probes. *Biol. Cell* **1990**, *70*, 159–165.
41. Le Guellec, D.; Frappart, L.; Desprez, P.Y. Ultrastructural localization of mRNA encoding for the EGF receptor in human breast cell cancer line BT20 by *in situ* hybridization. *J. Histochem. Cytochem*. **1991**, *39*, 1-6.
42. Le Guellec, D.; Trembleau, A.; Pechoux, C.; Gossard, F.; Morel, G. Ultrastructural non-radioactive *in situ* hybridization of GH mRNA in rat pituitary gland: Preembedding *vs.* ultrathin frozen sections vs postembedding. *J. Histochem. Cytochem.* **1992**, *40*, 979–986.
43. Morel, G.; Chabot, J.G.; Gossard, F.; Heisler, S. Is atrial natriuretic peptide synthesized and internalized by gonadotrophs? *Endocrinology* **1989**, *124*, 1703–1710.
44. Morel, G.; Dihl, F.; Gossard, F. Ultrastructural distribution of growth hormone (GH) mRNA and GH intron 1 sequences in rat pituitary gland: Effects of GH releasing factor and somatostatin. *Mol. Cell. Endocrinol.* **1989**, *65*, 81–90.
45. Pomeroy, M.E.; Lawrence, J.B.; Singer, R.H.; Billings-Gagliardi, S. Distribution of myosin heavy chain mRNA in embryonic muscle tissue visualized by ultrastructural *in situ* hybridization. *Dev. Biol.* **1991**, *143*, 58-67.
46. Singer, R.H.; Langevin, G.L.; Lawrence, J.B. Ultrastructural visualization of cytoskeletal mRNAs and their associated proteins using double-label *in situ* hybridization. *J. Cell. Biol.* **1989**, *108*, 2343–2353.
47. Trembleau, A.; Calas, A.; Fevre-Montange, M. Ultrastructural localization of oxytocin mRNA in the rat hypothalamus by *in situ* hybridization using a synthetic oligonucleotide. *Brain Res. Mol. Brain Res.* **1990**, *8*, 37–45.
48. Webster, H.F.; Lamperth, L.; Favilla, J.T.; Lemke, G.; Tesin, D.; Manuelidis, L. Use of a biotinylated probe and *in situ* hybridization for light and electron microscopic localization of Po mRNA in myelin-forming Schwann cells. *Histochemistry* **1987**, *86*, 441–444.
49. Wolber, R.A.; Beals, T.F.; Maassab, H.F. Ultrastructural localization of Herpes simplex virus RNA by *in situ* hybridization. *J. Histochem. Cytochem.* **1989**, *37*, 97–104.

50. Escaig-Haye, F.; Grigogiev, V.; Sharova, I.; Rudneva, V.; Buckrinskaya, A.; Fournier, J.G. Ultrastructural localization of HIV-1 RNA and core proteins. Simultaneous visualization using double immunogold labelling after *in situ* hybridization and immunocytochemistry. *J. Submicrosc. Cytol. Pathol.* **1992**, *24*, 437–443.
51. Egger, D.; Troxier, M.; Bienz, K. Light and electron microscopic *in situ* hybridization: Non-radioactive labeling and detection, double hybridization, and combined hybridization-immunocytochemistry. *J. Histochem. Cytochem.* **1994**, *42*, 815–822.
52. Gingras, D.; Bendayan, M. Colloidal gold electron microscopic *in situ* hybridization: Combination with immunocytochemistry for the study of insulin and amylase secretion. *Cell Vision* **1995**, *2*, 218–225.
53. Morey, A.L.; Ferguson, D.J.P.; Fleming, K.A. Combined immunocytochemistry and non-isotopic *in situ* hybridization for the ultrastructural investigation of human parvovirus B19 infection. *Histochem. J.* **1995**, *27*, 46–53.
54. Christian, H.C.; Taylor, A.D.; Flower, R.J.; Morris, J.F.; Buckingham, J.C. Characterization and localization of lipocortin 1-binding sites on rat anterior pituitary cells by fluorescence-activated cell analysis/sorting and electron microscopy. *Endocrinology* **1997**, *138*, 5341–5351.
55. Harper, C.V.; Featherstone, K.; Semprini, S.; Friedrichsen, S.; McNeilly, J.; Paszek, P.; Spiller, D.G.; McNeilly, A.S.; Mullins, J.J.; Davis, J.R.; *et al.* Dynamic organisation of prolactin gene expression in living pituitary tissue. *J. Cell Sci.* **2010**, *123*, 424–430.
56. Lacoste, T.D.; Michalet, X.; Pinaud, F.; Chemla, D.S.; Alivisatos, A.P.; Weiss, S. Ultrahigh-resolution multicolor colocalization of single fluorescent probes. *Proc. Natl. Acad. Sci. USA* **2000**, *97*, 9461–9466.
57. Michalet, X.; Pinaud, F.; Lacoste, T.D.; Dahan, M.; Bruchez, M.P.; Alivisatos, A.P.; Weiss, S. Properties of fluorescent semiconductor nanocrystals and their application to biological labeling. *Single Mol.* **2001**, *4*, 261-276.
58. Osamura, R.Y.; Egashira, N.; Yamazaki, M.; Miyai, S.; Takekoshi, S.; Kajiwara, H.; Kumai, N.; Umemura, S.; Yasuda, M.; Sanno, N.; *et al.* Mechanisms for production and secretion of hormones in physiologic and pathologic conditions. *Acta Histochem. Cytochem.* **2003**, *36*, 99–103.
59. Lledo, P.M.; Vernier, P.; Vincent, J.D.; Mason, W.T.; Zorec, R. Inhibition of Rab3B expression attenuates Ca(2+)-dependent exocytosis in rat anterior pituitary cells. *Nature* **1993**, *364*, 540–544.
60. Mizoguchi, A. Rab3A-RabGDI-Rabphilin-3A system regulating membrane fusion machinery in the synapse and the growth cone. *Acta Histochem. Cytochem.* **1994**, *27*, 117–126.
61. Tasaka, K.; Masumoto, N.; Mizuki, J.; Ikebuchi, Y.; Ohmichi, M.; Kurachi, H.; Miyake, A.; Murata, Y. Rab3B is essential for GnRH-induced gonadotrophin release from anterior pituitary cells. *J. Endocrinol.* **1998**, *157*, 267–274.
62. Tahara, S.; Sanno, N.; Teramoto, A.; Osamura, R.Y. Expression of Rab3, a Ras-related GTP-binding protein, in human nontumorous pituitaries and pituitary adenomas. *Mod. Pathol.* **1999**, *12*, 627–634.
63. Hess, D.T.; Slater, T.M.; Wilson, M.C.; Skene, J.H. The 25 kDa synaptosomal-associated protein SNAP-25 is the major methionine-rich polypeptide in rapid axonal transport and a major substrate for palmitoylation in adult CNS. *J. Neurosci*. **1992**, *12*, 4634–4641.

64. Oyler, G.A.; Higgins, G.A.; Hart, R.A.; Battenberg, E.; Billingsley, M.; Bloom, F.E.; Wilson, M.C. The identification of a novel synaptosomal-associated protein, SNAP-25, differentially expressed by neuronal subpopulations. *J. Cell Biol.* **1989**, *109*, 3039–3052.
65. Bennett, M.K.; Calakos, N.; Scheller, R.H. Syntaxin: A synaptic protein implicated in docking of synaptic vesicles at presynaptic active zones. *Science* **1992**, *257*, 255–259.
66. Calakos, N.; Bennett, M.K.; Peterson, K.E.; Scheller, R.H. Protein-protein interactions contributing to the specificity of intracellular vesicular trafficking. *Science* **1994**, *263*, 1146–1149.
67. Sollner, T.; Bennett, M.K.; Whiteheart, S.W.; Scheller, R.H.; Rothman, J.E. A protein assembly-disassembly pathway *in vitro* that may correspond to sequential steps of synaptic vesicle docking, activation, and fusion. *Cell* **1993**, *75*, 409–418.
68. Sollner, T.; Whiteheart, S.W.; Brunner, M.; Erdjument-Bromage, H.; Geromanos, S.; Tempst, P.; Rothman, J.E. SNAP receptors implicated in vesicle targeting and fusion. *Nature* **1993**, *362*, 318-324.
69. Garcia, E.P.; Gatti, E.; Butler, M.; Burton, J.; De Camilli, P. A rat brain Sec1 homologue related to Rop and UNC18 interacts with syntaxin. *Proc. Natl. Acad. Sci. USA* **1994**, *91*, 2003–2007.
70. Hata, Y.; Slaughter, C.A.; Sudhof, T.C. Synaptic vesicle fusion complex contains unc-18 homologue bound to syntaxin. *Nature* **1993**, *366*, 347–351.
71. Pevsner, J.; Hsu, S.C.; Scheller, R.H. n-Sec1: A neural-specific syntaxin-binding protein. *Proc. Natl. Acad. Sci. USA* **1994**, *91*, 1445–1449.
72. Pevsner, J.; Hsu, S.C.; Braun, J.E.; Calakos, N.; Ting, A.E.; Bennett, M.K.; Scheller, R.H. Specificity and regulation of a synaptic vesicle docking complex. *Neuron* **1994**, *13*, 353–361.
73. Jacobsson, G.; Meister, B. Molecular components of the exocytotic machinery in the rat pituitary gland. *Endocrinology* **1996**, *137*, 5344–5356.
74. Salinas, E.; Quintanar, J.L.; Reig, J.A. Immunohistochemical study of Syntaxin-1 and SNAP-25 in the pituitaries of mouse, guinea pig and cat. *Acta Physiol. Pharmacol. Ther. Latinoam.* **1999**, *49*, 61–64.
75. Quintanar, J.L.; Salinas, E. Effect of hypothyroidism on synaptosomal-associated protein of 25 kDa and syntaxin-1 expression in adenohypophyses of rat. *J. Endocrinol. Invest.* **2002**, *25*, 754–758.
76. Tsien, R.Y. The green fluorescent protein. *Annu. Rev. Biochem.* **1998**, *67*, 509–544.
77. Magoulas, C.; McGuinness, L.; Balthasar, N.; Carmignac, D.F.; Sesay, A.K.; Mathers, K.E.; Christian, H.; Candeil, L.; Bonnefont, X.; Mollard, P.; *et al.* A secreted fluorescent reporter targeted to pituitary growth hormone cells in transgenic mice. *Endocrinology* **2000**, *141*, 4681-4689.
78. Lee, E.J.; Duan, W.R.; Kotlar, T.; Jameson, J.L. Restoration of growth hormone-releasing hormone (GHRH) responsiveness in pituitary GH3 cells by adenovirus-directed expression of the human GHRH receptor. *Endocrinology* **2001**, *142*, 414–420.
79. He, Z.; Fernandez-Fuente, M.; Strom, M.; Cheung, L.; Robinson, I.C.; Le Tissier, P. Continuous on-line monitoring of secretion from rodent pituitary endocrine cells using fluorescent protein surrogate markers. *J. Neuroendocrinol.* **2011**, *23*, 197–207.

© 2011 by the authors; licensee MDPI, Basel, Switzerland. This article is an open access article distributed under the terms and conditions of the Creative Commons Attribution license (http://creativecommons.org/licenses/by/3.0/).
